# Assessment of efficacy and safety of UV-based therapy for psoriasis: a network meta-analysis of randomized controlled trials

**DOI:** 10.1080/07853890.2021.2022187

**Published:** 2022-01-06

**Authors:** Yajia Li, Ziqin Cao, Jia Guo, Qiangxiang Li, Wu Zhu, Yehong Kuang, Xiang Chen

**Affiliations:** a Department of Dermatology, Hunan Key Laboratory of Skin Cancer and Psoriasis; Hunan Engineering Research Center of Skin Health and Disease, Xiangya Hospital, Central South University, Changsha, China; b Department of Spine Surgery, the Xiangya Hospital, Central South University, Changsha, Hunan, China; c National Clinical Research Center for Geriatric Disorders of Xiangya hospital, Central South University (Sub-center of Ningxia), Yinchuan, Ningxia Hui Autonomous Region 750001, China; d Ningxia Geriatric Disease Clinical Research Center, People's Hospital of Ningxia Hui Autonomous Region, Yinchuan, Ningxia Hui Autonomous Region 750001, China; e Hunan People's Hospital, Geriatrics Institute of Hunan Province, Changsha, China, Changsha 410002, China; f National Clinical Research Center for Geriatric Disorders, Xiangya Hospital, Central South University, Changsha, China

**Keywords:** Psoriasis; phototherapy; UV-based therapy; network meta-analysis

## Abstract

**Background:**

Previous studies have proven that ultraviolet (UV)-based phototherapy, including UVB or psoralen UVA (PUVA), and their combination therapies, is effective for psoriasis treatment.

**Objective:**

To compare the clinical efficacy and adverse events (AEs) of different UV-based phototherapy in psoriasis.

**Methods:**

PubMed, Cochrane Library, Scopus and Embase were systematically searched. A random-effect model network meta-analysis with frequentist framework was performed, and results were reported as risk ratios (RRs) with 95% CI. The main variable for assessing effectiveness and safety are PASI 75 response and withdrawal due to AEs. Ranking effects were calculated by surface under the cumulative ranking analysis (SUCRA).

**Results:**

Thirty-two studies involving a total of 2120 psoriasis patients were included in this network meta-analysis. Overall, no significant difference was reported with respect to withdrawal due to AEs or incidence of erythema. The relatively safest strategy was combined adjuvant therapy with PUVA (cPUVA), especially PUVA combined with calcium/vitamin D derivatives (RR 0.98, 95% CI [0.30–3.17], SUCRA = 80.8%). Both cPUVA (RR 1.39, 95% CI [1.00– 1.94]) and combined adjuvant therapy with UVB (cUVB) (RR 1.27, 95% CI [1.03–1.57]) showed a superior effect than the monotherapy of UVA or UVB, respectively. PUVA combined with vitamin D and its derivatives (PAVD) ranked highest concerning clinical effect and safety (clusterank value = 7393.2).

**Conclusions:**

The efficacy of all the combination therapy regimens was significantly superior to that of UV monotherapy, without significant differences in tolerability and safety. cUVB and cPUVA, and particularly the combination of UVA with calcium/vitamin D derivatives, was ranked as the overall safest and most effective phototherapy method.

## 1. Introduction

Psoriasis is known as a common, immune-related, chronic inflammatory skin disorder and the estimated prevalence was ranging from 0.51 to 11.4% in worldwide adults [1]. Plaque psoriasis is the most common type, and most psoriasis patients suffering from the mild-to-moderate disease can be treated with topical treatments, whereas severe cases require additional therapeutic options [2]. Systemic therapies, including oral retinoids, as well as biologics or phototherapy are used for the long-term treatment of psoriasis [3]. Ultraviolet (UV)-based phototherapy with UVB or psoralen UVA (PUVA) is also a well-established and generally effective treatment for chronic plaque psoriasis employing UV light with or without photosensitizers [4].

Although systemic and biological treatments are strongly recommended for severe and diffuse skin diseases, these medications can determine systemic side effects and immunosuppression. Based on the evidence from National Psoriasis Foundation, UVB phototherapy (broadband [BB] or narrow-band [NB]) is suggested for patients suffering from moderate-to-severe psoriasis before adopting treatments with systemic and biologic agents. Besides, PUVA is indicated for patients with poor NB-UVB control and high psoriasis area and severity index (PASI) scores [5]. Among phototherapy regimens for psoriasis, broadband UVB (BB-UVB) has the longest history and narrowband UVB (NB-UVB) represents the first-line phototherapy treatment, also in combination with other regimens [6]. These UV-based phototherapies, along with their combination with other treatments, are thought to be effective treatments for many psoriasis patients, leading to an urgent demand for comparative studies of efficacy referring to clinical decision making. A meta-analysis published in 2013 comparing the efficacy of localized phototherapies showed that topical PUVA treatment is more effective than non-laser targeted UVB phototherapy [7]. Almutawa et al. summarized characteristics of UV-based therapies, indicating that PUVA monotherapy showed higher effectiveness than UVB-based therapy in determining complete skin clearance of patients with moderate-to-severe plaque-type psoriasis [8]. Since then, there have been several randomized controlled trials (RCTs) on demonstrating and comparing the efficacy, safety or tolerability of monotherapies and UV-based combinatorial therapies. However, no comprehensive studies evaluating relative UV efficacy and tolerability have been carried out so far. Although several head-to-head trials exist, RCTs have not considered all possible pairwise combinations of UV-based therapies with agents, or simultaneous direct comparisons of multiple treatments.

In this study, we performed an NMA collecting direct and indirect evidence from several RCTs simultaneously [9]. Specifically, the achievement of PASI 75 response (75% or more reduction in PASI score from baseline) in the short-term period of treatment was chosen to measure its efficacy since it is indicative of both severities and involved area of psoriatic lesions, as well as it is evaluated and described in most reported trials and reviews [10,11]. This study was aimed to compare the efficacy and tolerability of UV-based therapies, used as monotherapy or in combination with various drugs and remedies, including systematic treatments, skin lubricants, vitamins and vitamin derivatives, for the treatment of moderate-to-seve*r*e psoriasis. We also considered the absolute effects of therapies to provide information supporting clinical decision-making and care planning.

## 2. Materials and methods

The study protocol was registered in the international prospective register of systematic reviews (https://www.crd.york.ac.uk/PROSPERO), number CRD42021232906. The research question in the manuscript was how different UV-based phototherapies impacted the efficacy and safety for the treatment of psoriasis.

### 2.1. Data sources and searches

We designed and conducted this study following the Preferred Reporting Items for Systematic Reviews and Meta-Analyses (PRISMA) guideline [12].

Two authors (Y.L. and Z.C.) independently and systematically searched the following queries in PubMed, Cochrane Library, Scopus and Embase, from January 1980 to January 2021: “psoriasis” OR “psoriatic” AND “light therapy” OR “phototherapy” and “ultraviolet radiation A” OR “UVA” OR “ultraviolet radiation A with psoralen” OR “PUVA” OR “ultraviolet radiation B” OR “UVB” OR “excimer” OR “TL-01” OR “PUVA” AND “combination” OR “UVB” AND “combination”. Additional potentially eligible studies were screened by further reviewing reference lists of identified articles. There was no restriction on the language of publication.

### 2.2. Study selection

The following studies were used in our NMA: 1. studies analysing psoriasis patients undergoing phototherapy; 2. studies comparing two or more different treatment strategies; 3. studies describing RCTs with prospective parallel-group design; 4. studies reporting at least one of the following outcomes: PASI, withdrawal due to adverse events (AEs), and incidence of erythema. On the contrary, we excluded: 1. low-quality studies according to the corresponding quality assessment tools; 2. dose-escalation studies made using only one treatment strategy; 3. animal studies, *in vitro* biomechanical studies, cadaver studies, case-control studies, reviews, systematic reviews and meta-analyses, conference abstracts, letters and analyses not comprising original data.

For studies with insufficient data, we contacted the corresponding authors. We chose to contact the corresponding authors when relatively to data of studies were presented as figures and not as numeric data in the manuscript text or tables. If no response was received, two current authors (Y.L. and Z.C.) independently extrapolated data from the graphs/figures. If that was not possible the study was excluded. All disagreements were resolved according to the discussion with the third author (J.G.).

### 2.3. Data extraction and quality assessment

Next, the qualities of the included studies were assessed by evaluating the methodological quality and the bias risk of RCTs, calculated by using the Cochrane risk of the bias assessment tool [13]. The following six indices were evaluated and divided into unclear, low or high risk of bias, including sequence generation, allocation concealment, blinding, incomplete outcome data, selection outcome reporting and other sources of bias.

We extracted the relevant data from studies, including the first author, year of publication, the number of participants, mean age, gender ratio, type or degree of psoriasis, mean of follow-up and outcomes data. The data in the intention-to-treat analysis were preferred for avoiding the influence of withdrawal bias if present.

### 2.4. Outcome measures

The primary efficacy endpoints were the percentage of patients achieving PASI 75 or a PASI response greater than PASI 75 if the latter was not available. As for the other assessment standards, we only included trials reporting the complete clearance of the disease or nearly clearance (90% improvement from the baseline). Those trials that reported PASI 75, PASI 90 or clearance were grouped into PASI 75 and above.

Considering that the differences between the baseline values of each included study may reduce the reliability of the results and conclusions, the changes from the baseline value of PASI (PASI improvement) at the last follow-up were calculated (mean ± SD) and used to reconfirm the findings from PASI 75 or above. For studies not reporting PASI improvement, the correlation coefficient method recommended by Cochrane Handbook [13] was used to calculate the changes.

Considering the impact of patient compliance on the treatment effect in clinical practice, withdrawal due to AEs was chosen as the primary safety endpoint, and to estimate treatment tolerability. The secondary safety endpoint was erythema, the most common AEs related to UV-based therapies. The risk ratio (RR) with 95% CI was used to compare the relative safety of treatments.

### 2.5. Statistical analysis

Random-effects NMA were conducted by using Stata/MP software version 14.0 (Stata Corp, College Station, TX) with a frequentist framework.

The proportional variance-covariance matrix data were pooled using the random-effects multivariate meta-regression, whereas the model fit was evaluated by the restricted maximum-likelihood method [14].

Inconsistencies were assessed with global inconsistency tests and node-split tests, and only if both results reported no significant inconsistency (*p* > .05), the consistency model was adopted. If inconsistency was reported in any network, a sensitivity analysis was used to identify the source of inconsistency and to exclude studies from the network. Publication biases for each network were evaluated by funnel plots, and for networks whose funnel plots showed possible asymmetry, the presence of publication bias was assessed by the egger’s test. The relative efficacy and safety of different phototherapy strategies were ranked using surface under the cumulative ranking (SUCRA) probabilities [15] and cluster-ranking plots were constructed to determine the optimal choice for multiple outcome indicators. Further subgroup analysis was conducted for exploring the optimal combination of drugs. Data relative to four subgroups of drugs, including vitamin A and its derivatives, vitamin D and its derivatives, skin lubricants, and system treatments, were obtained.

Differences between treatments were considered significant when the 95% CI did not contain 1 for RR or 0 for SMD and WMD. *p* < .05 was considered as significant level in statistics.

## 3. Results

### 3.1. Literature selection

**Figure 1.**
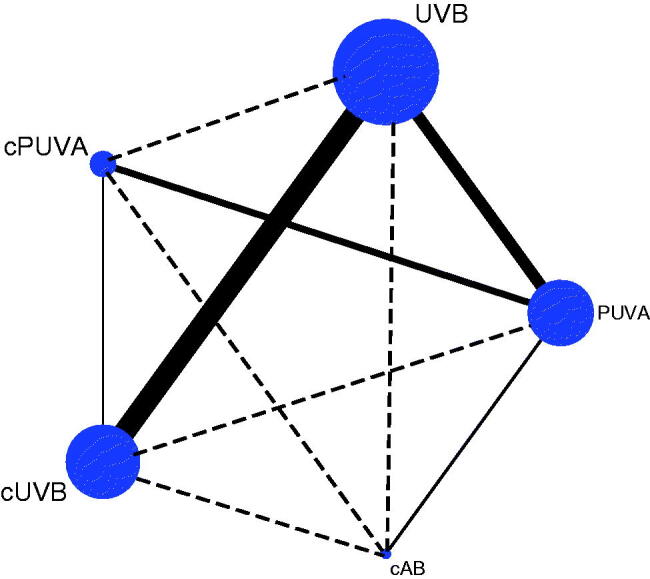
Structure of network formed by interventions. The lines between treatment nodes indicate the direct comparisons made within randomized controlled trials. The solid line means direct comparisons and the dashed line means indirect comparisons. (A) Main network meta-analysis. (B) Subgroup analysis.

Thirty-two studies were included following the systematic screening of the Literature (Figure S1) [16–47]. In the main network analysis, five different phototherapy strategies were identified and analysed: PUVA, UVB, combined adjuvant therapy with PUVA (cPUVA), combined adjuvant therapy with UVB (cUVB) and the combination of PUVA with UVB (cAB). According to the category and activity mechanism of the adjuvant treatment, nine subgroups were identified and analysed: PUVA, UVB, PAVA (PUVA combined with vitamin A and its derivatives), PAVD (PUVA combined with vitamin D and its derivatives), UBVA (UVB combined with vitamin A and its derivatives), UBVD (UVB combined with vitamin D and its derivatives), UBST (UVB combined with systematic treatments), UBSL (UVB combined with skin lubricants) and cAB. The network plot of the main network analysis and subgroup analysis are shown in Figure 1.

### 3.2. Study characteristics

The analysis involved a total of 2120 psoriasis patients. The median age of subjects was 41.5 years (interquartile distance 37.32 − 45.00), and the median percentage of male patients was 61.6% (ranging 35.71–100.00%) (Table S1). Details on quality and bias-risk assessments of all studies are shown in Table S2. The funnel plots of the main network meta-analysis and subgroup analysis are shown in Figures S1 and S2, respectively.

**Table 1.** Detailed results of main network analysis.

Treatment	RR (95% CI) for PASI 75 response	SUCRA for PASI 75 response, %	RR (95% CI) for PASI 75 and above response	SUCRA for PASI 75 and above response, %	SMD (95% CI) for absolute PASI improvement	SUCRA for absolute PASI improvement, %	RR (95% CI) for withdrawal due to AEs	SUCRA for withdrawal due to AEs, %	RR (95%CI) for incidence of erythema	SUCRA for incidence of erythema, %
PUVA	1.06 (0.65, 1.71)	26.9	0.94 (0.66, 1.34)	23.5	1.41 (−2.13, 4.96)	19.4	Reference	46.6	Reference	39.1
UVB	1.09 (0.63, 1.88)	32.0	0.94 (0.64, 1.38)	23.3	0.46 (−3.02, 3.95)	23.3	0.95 (0.34, 2.68)	49.3	0.89 (0.69, 1.16)	65.8
cPUVA	1.47 (0.82, 2.64)	86.0	1.15 (0.77, 1.73)	81.1	−0.76 (−4.10, 2.58)	89.5	1.00 (0.29, 3.51)	46.7	0.95 (0.59, 1.52)	52.6
cUVB	1.38 (0.77, 2.47)	79.0	1.13 (0.76, 1.68)	78.4	−0.89 (−3.93, 2.15)	67.4	0.87 (0.23, 3.31)	54.9	0.90 (0.61, 1.32)	61.0
cAB	Reference	26.1	Reference	43.8	Reference	50.4	0.83 (0.02, 41.33)	52.5	1.20 (0.44, 3.25)	31.5

**Table 2.** The league plots of main network meta-analysis (from the top left to the bottom right, higher comparator *vs.* lower comparator).

a. PASI 75 and above response (lower left) and PASI 75 response (upper right), RR with 95% CI.				
cPUVA	0.94 (0.64–1.38)	0.74 (0.51–1.07)	0.72 (0.52–1.00)	0.68 (0.38–1.22)
1.02 (0.79–1.31)	cUVB	0.79 (0.64–0.97)	0.77 (0.55–1.06)	0.72 (0.40–1.30)
1.22 (0.97–1.55)	1.20 (1.06–1.37)	UVB	0.97 (0.75–1.26)	0.92 (0.53–1.59)
1.23 (1.01–1.50)	1.20 (0.99–1.46)	1.00 (0.87–1.15)	PUVA	0.94 (0.58–1.53)
1.15 (0.77–1.73)	1.13 (0.76–1.68)	0.94 (0.64–1.38)	0.94 (0.66–1.34)	cAB
				

b. Absolute PASI improvement (lower left), SMD with 95% CI.				
cPUVA				
0.95 (−1.11–3.01)	cUVB	–	–	–
2.17 (0.15–4.18)	1.22 (0.05–2.39)	UVB	–	–
2.31 (0.48–4.13)	1.35 (−0.35–3.06)	0.14 (−1.25–1.52)	PUVA	–
1.41 (−2.13–4.96)	0.46 (−3.02–3.95)	−0.76 (−4.10–2.58)	−0.89 (−3.93–2.15)	cAB

c. Withdrawal due to AEs (lower left) and incidence of erythema (upper right), RR with 95% CI.				
cUVB	1.05 (0.61–1.80)	1.33 (0.46–3.87)	0.99 (0.74–1.34)	1.11 (0.75–1.63)
0.87 (0.15–4.89)	cPUVA	1.27 (0.42–3.83)	0.95 (0.57–1.57)	1.06 (0.66–1.70)
1.05 (0.02–65.78)	1.21 (0.02–73.81)	cAB	0.75 (0.27–2.09)	0.83 (0.31–2.26)
0.91 (0.37–2.26)	1.05 (0.22–5.00)	0.87 (0.02–49.62)	UVB	1.12 (0.86–1.45)
0.87 (0.23–3.31)	1.00 (0.29–3.51)	0.83 (0.02–41.33)	0.95 (0.34–2.68)	PUVA

### 3.3. Main network meta-analysis

#### 3.3.1. PASI 75 response

A total of 19 trials with 1476 patients were included in this network. No inconsistency was detected after the application of the consistency model. Based on the SUCRA values, the cPUVA group obtained the highest PASI 75 response (SUCRA = 86.0%), followed by cUVB (SUCRA = 79.0%) and UVB (SUCRA = 32.0%), whereas the lowest PASI 75 was achieved following CAB treatment (SUCRA = 26.1%). The cPUVA group was numerically higher than PUVA group (RR 1.39, 95% CI [1.00–1.94]), whereas the cUVB group outnumbered the UVB group (RR 1.27, 95% CI [1.03–1.57]).

#### 3.3.2. PASI 75 and above response

A total of 32 trials involving 2120 patients were included in this network. No significant inconsistencies were found in applying the consistency model. Similar to PASI 75 response results, cPUVA and cUVB determined a better response in patients (SUCRA = 81.1 and 78.4%, respectively), whereas UVB was the treatment less effective (SUCRA = 23.3%). Significant differences were only found between cPUVA- and PUVA-treated groups (RR 1.23, 95% CI [1.01–1.50]), cUVB and UVB (RR 1.20, 95% CI [1.06–1.37]).

#### 3.3.3. PASI improvement

For this network, we only analysed data on 844 patients in 14 trials, so these results should be explained with caution. No significant inconsistency was detected by using the consistency model. Although the funnel plot of this network showed dubious asymmetry, egger’s tests did not report any significant publication bias or small-study effect (*p* = .661). From these analyses, the cPUVA group resulted to respond significantly better than PUVA (SMD 2.31, 95% CI [0.48–4.13]) or UVB (SMD 2.17, 95% CI [0.15–4.18]) groups, and the cUVB-treated group achieved better PASI responses than UVB-treated group (SMD 1.22, 95% CI [0.05–2.39]). According to SUCRA, cPUVA and cUVB ranked highest in efficacy (SUCRA = 89.5 and 67.4%, respectively), whereas PUVA was the lowest-ranked treatment (SUCRA = 19.4%).

#### 3.3.4. Safety outcomes

Networks relative to 23 trials (1410 patients) and 20 trials (1361 patients) reporting withdrawal due to AEs, and incidence of erythema, respectively were assessed. The consistency model application did not reveal inconsistencies for the two analysed networks.

No significant difference was reported relative to withdrawal due to AEs. The PUVA group showed the highest withdrawal rate according to SUCRA (reference, SUCRA = 46.6%), while cUVB (RR 0.87, 95% CI [0.23–3.31], SUCRA = 54.9%) and cAB (RR 0.83, 95% CI [0.02–41.33], SUCRA = 52.5%) showed the lowest rate.

**Figure 2.**
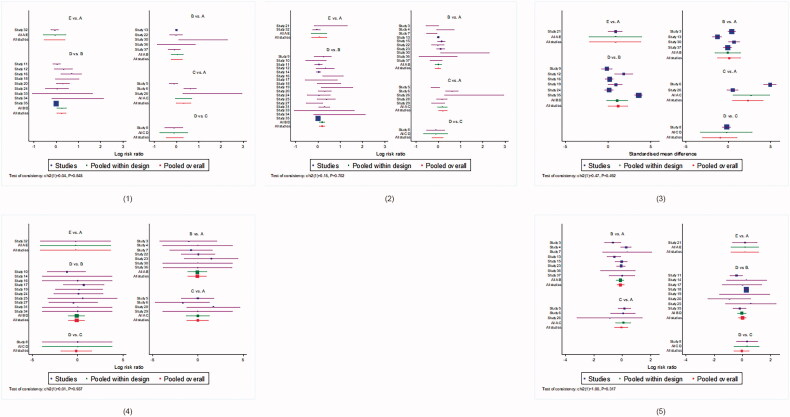
Forest plots incorporated direct comparisons and indirect comparisons of main network meta-analysis. (1) PASI 75 response. (2) PASI 75 and above response. (3) Absolute PASI improvement. (4) Withdrawal due to AEs. (5) Incidence of erythema. (A: PUVA; B: UVB; C: cPUVA; D: cUVB; E: cAB).

**Figure 3.**
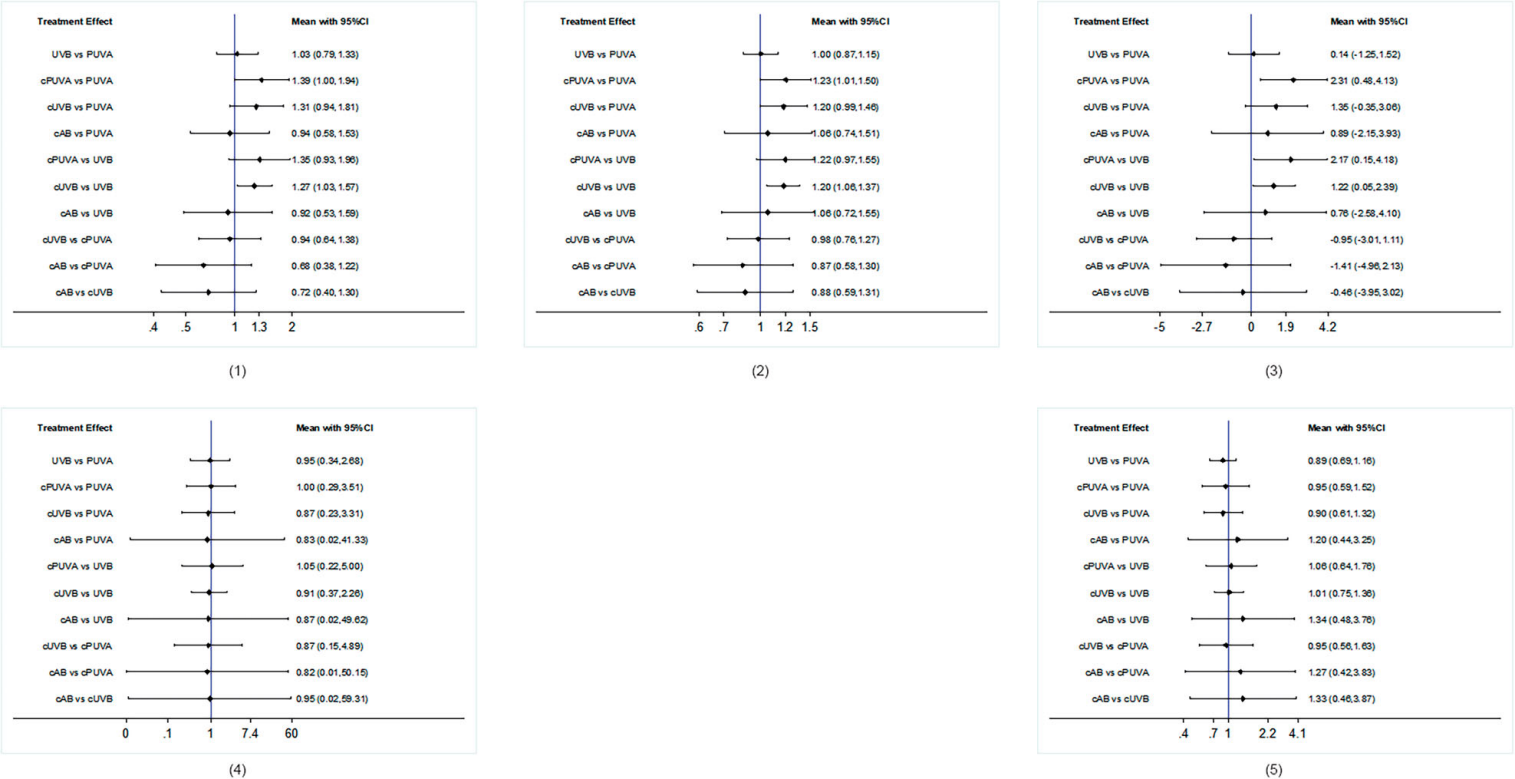
Forest plots of network comparisons of main network meta-analysis. (1) PASI 75 response. (2) PASI 75 and above response. (3) Absolute PASI improvement. (4) Withdrawal due to AEs. (5) Incidence of erythema.

No significant difference was also reported for the incidence of erythema. Based on the SUCRA ranking,

UVB and cUVB showed less incidence of erythema (RR 0.75 95% CI [0.27–2.09], SUCRA = 65.8% and RR 0.75, 95% CI [0. 26–2.19], SUCRA = 61.0%, respectively), whereas cAB group showed the worst response (reference, SUCRA = 31.5%). SUCRA values of main network analyses are shown in Table 1, and the forest plots in Figures 2 and 3.

According to the cluster-rank results, cUVB and cPUVA ranked highest with respect to effect (PASI 75 response) and safety (withdrawal due to AEs) combined parameters (clusterank value = 4337.1 and 4016.2, respectively), whereas PUVA ranked lowest (clusterank value = 1253.5) (Figure S4). In Table 2, the league plots relative to differences between network meta-analysis groups are shown.

### 3.4. Subgroup analysis

#### 3.4.1. PASI 75 response

A total of 20 trials with 1500 patients were included in this network. The application of the consistency model did not reveal any inconsistency. PAVD treatment was potentially the best therapeutical strategy according to SUCRA (SUCRA = 91.5%), followed by UBST (SUCRA = 83.0%) and UBSL (SUCRA = 54.5%) therapies. UVB was the less effective treatment (SUCRA = 24.8%). In addition, PAVD was sìgnificantly better than both PUVA (RR 1.86, 95% CI [1.15–3.01]) and UVB (RR 1.87, 95% CI [1.09–3.19]) treatments, and UBST was more efficacious than UVB (RR 1.58, 95% CI [1.08–2.31]).

#### 3.4.2. PASI 75 and above response

A total of 32 trials with 2120 patients were involved in this network. Also for this analysis, no significant inconsistency was found. PAVD and UBST groups showed a better response, in terms of achievement of a PASI 75 and above response (SUCRA = 97.4 and 70.8%), and, similarly to what was observed for PASI 75 response network, UVB ranked lowest (SUCRA = 18.2%). PAVD treatment determined a better response than PAVA (RR 1.69, 95% CI [1.05–2.72]), UBSL (RR 1.72, 95% CI [1.04–2.84]), cAB (RR 1.78, 95% CI [1.04–3.03]), PUVA (RR 1.86, 95% CI [1.22–2.84]) and UVB (RR 1.88, 95% CI [1.21–2.92]) therapies. UBST was also better than UVB treatment (RR 1.26, 95% CI [1.01–1.57]).

#### 3.4.3. PASI improvement

Fourteen trials with 844 patients could be included in this network. Although the application of the egger’s test revealed no concern (*p* = .668), the publication bias and small-study effect could likely represent critical issues. Again, the consistency model was adopted. PAVD- and UBSL-treated patients showed a better response as compared to patients undergone the other treatments. According to SUCRA, PAVD and UBSL ranked highest (SUCRA = 98.3 and 88.9%, respectively), whereas UVB ranked lowest (SUCRA = 18.6%).

#### 3.4.4. Safety outcomes

Two networks relative to 23 trials (1410 patients) reporting the withdrawal due to AEs and 20 trials (1361 patients) reporting the incidence of erythema, respectively, were assessed. No significant inconsistencies were detected using the consistency model.

No significant difference was reported with respect to withdrawal due to AEs or incidence of erythema. In line with SUCRA result of withdrawal rate, PAVA and PAVD were the worst- and the best-tolerated treatments (reference, SUCRA = 32.2% and RR 0.98, 95% CI [0.30–3.17], SUCRA = 80.8%, respectively). UBVA group reported the highest incidence of erythema (reference, SUCRA = 23.2%), whereas UBVD the lowest (RR 0.42, 95% CI [0.12–1.51], SUCRA = 83.6%).

According to the cluster-rank results, PAVD and UBST ranked highest concerning clinical effect (PASI75 response) and safety (withdrawal due to AEs) (clusterank value = 7393.2 and 5419.9, respectively), whereas UVB ranked lowest (clusterank value = 1205.3) (Figure S5). SUCRA values are shown in Table 3, and forest plots of the subgroup analysis are reported in Figures S6 and S7. The league plots of subgroup analysis are presented in Tables S3–S5.

**Table 3.** Detailed results of subgroup analysis.

Treatment	RR (95% CI) for PASI 75 response	SUCRA for PASI 75 response, %	RR (95% CI) for PASI 75 and above response	SUCRA for PASI 75 and above response, %	SMD (95% CI) for absolute PASI improvement	SUCRA for absolute PASI improvement, %	RR (95% CI) for withdrawal due to AEs	SUCRA for withdrawal due to AEs, %	RR (95% CI) for incidence of erythema	SUCRA for incidence of erythema, %
PUVA	1.00 (0.79, 1.28)	26.5	1.01 (0.88, 1.15)	21.1	0.07 (-0.70,0.83)	22.5	0.92 (0.49,1.73)	46.7	0.92 (0.49,1.73)	43.6
UVB	Reference	24.8	Reference	18.2	Reference	18.6	0.80 (0.41,1.57)	48.6	0.80 (0.41,1.57)	62.7
PAVA	1.14 (0.73, 1.78)	45.4	1.11 (0.87, 1.41)	45.1	0.73 (−0.63, 2.09)	49.4	Reference	32.2	Reference	39.8
PAVD	1.87 (1.09, 3.19)	91.5	1.88 (1.21, 2.92)	97.4	4.97 (3.12, 6.82)	98.3	0.98 (0.30, 3.17)	80.8	0.98 (0.30, 3.17)	44.1
UBVA	1.19 (0.75, 1.89)	51.5	1.21 (0.89, 1.64)	60.2	0.67 (−0.43, 1.77)	48.3	1.37 (0.57, 3.33)	40.4	1.37 (0.57, 3.33)	23.2
UBVD	1.04 (0.73, 1.48)	34.0	1.19 (0.96, 1.49)	60.2	0.13 (−1.47, 1.73)	27.9	0.58 (0.23, 1.46)	42.8	0.58 (0.23, 1.46)	83.6
UBST	1.58 (1.08, 2.31)	83.0	1.26 (1.01, 1.57)	70.8	0.51 (−0.66, 1.68)	42.6	0.68 (0.21, 2.15)	65.3	0.68 (0.21, 2.15)	68.3
UBSL	1.21 (0.81, 1.80)	54.4	1.09 (0.87, 1.37)	41.5	3.60 (2.03, 5.18)	88.9	0.88 (0.40, 1.92)	42.8	0.88 (0.40, 1.92)	49.3
cAB	1.08 (0.69, 1.69)	39.0	1.05 (0.74, 1.50)	35.4	0.96 (−0.93, 2.85)	53.5	1.11 (0.34, 3.57)	50.5	1.11 (0.34, 3.57)	35.2

## 4. Discussion

We conducted the first network meta-analysis based on high-quality RCTs comprehensively comparing the effects and tolerability of UV-based phototherapy strategies for the moderate-to-severe psoriasis treatments. This NMA represents a valuable comparison of all currently licenced or commonly used UVB or PUVA phototherapies and their combinations with other treatments.

Our findings are largely in accordance with and based on evidence from those previously published guidelines and systematic reviews. A previous systematic literature review by Archier et al. [48] showed that although PUVA tended to clear plaque-type psoriasis more efficiently than NB-UVB, dermatologists prefer the latter treatment as the first-line phototherapy, due to cutaneous carcinogenic risk by PUVA and the easier administration procedure of UVB. In parallel, a meta-analysis by Almutawa et al. reported that topical PUVA was superior to non-laser-targeted UVB phototherapy in terms of effectiveness [7]. However, though we found the PUVA group showed the highest withdrawal rate according to SUCRA, there was no significant difference when comparing PUVA with UVB treatments in terms of efficacy and safety. This discrepancy might be due to the lack of sufficient studies summarizing all kinds of adverse effects, such as pruritus, nausea, vertigo, headache, etc. The information available in the analysed studies permitted only to conclude that both groups developed cutaneous erythematous reaction, which is an adverse effect particularly common in phototherapy. In our opinion, the severe side-effects of PUVA observed more frequently in the PUVA group could not be ignored. Markham et al. [35] also reported that NB-UVB (TL-01) had fewer side effects than PUVA in treatments of patients with chronic plaque psoriasis. Interestingly, the efficacy of all the combination therapy regimens was significantly higher than that observed using UV in monotherapy. cPUVA also determined a more substantial improving skin lesions without a lower safety than PUVA Therefore, cPUVA could be used for moderate-to-severe psoriasis cases as an alternative to UVB therapies if ineffective. We also found that when compared with PAVA, PUVA, UVB and CAB, the PUVA therapies administered together with calcium/vitamin D derivatives, such as calcipotriene, showed higher efficacy and were more tolerated than single treatments. Consistently, a recent meta-analysis showed that targeted UVB combined with calcipotriene was more efficacious than targeted UVB alone, and the efficacy of combination therapy could be related to the analogous and complementary roles associated with calcipotriene on UVB [49]. Calcipotriol cream is used to treat plaque psoriasis and is generally preferred to the ointment formulation as it is less greasy. UVB and Calcipotriene can increase the level of 1,25-dihydroxy vitamin D3 and inhibit the proliferation of the epidermal cell. Therapeutic effects of vitamin D3 analogues persist after PUVA or NB-UVB irradiation, and calcipotriene ointment addition to PUVA determines a UVA sparing effect as well as clearing of psoriatic lesions with low UVA accumulation [50,51]. Combination of PUVA with calcipotriene reduces the UVA dosage required for clearance of psoriasis, thus decreasing the long-term risk of cutaneous malignancy and increasing the efficacy of treatment. Our study could not produce corresponding data on the accumulated dose required for clearance, and more clinical studies are necessary to obtain this evidence.

Importantly, both the outcomes of efficacy and safety were considered together in cluster-ranking plots and joint rankings and provided absolute effect estimates to help clinical decision making: (1) The efficacy of all the combination therapy regimens was significantly higher than that of UV monotherapy, and there was no significant difference in their tolerability and safety; (2) cPUVA showed an optimal efficacy to improve skin lesions, especially when combined with calcium/vitamin D derivatives to treat moderate-to-severe psoriasis; (3) PUVA and UVB treatments have no significant differences in efficacy and safety, nor the combination of PUVA with UVB increase efficacy. On the contrary, PUVA and UVB co-treatment increased the risk of erythema according to SUCRA results; (4) PAVD regimen showed higher efficacy compared with PAVA, PUVA, UVB, and CAB treatments, as well as it was the best-tolerated so that it could have the potential to be the optimal choice in the clinical decision; (5) based on cluster-rank analysis, cPUVA (PAVD) and cUVB (UBST) are the safest and most effective phototherapy methods to treat psoriasis.

On the other hand, our study had some limitations. Although observational studies and other non-randomized control trials (nRCTs) also play an indispensable role in evaluating the effectiveness and safety, especially the long-term effects, of therapy, we only included high-quality RCTs to avoid the unmanageable confounding factors existing in observational studies and other nRCTs. This may be one of the reasons for the relatively insufficient number of articles included in this study. Therefore, publication bias represented a non-negligible problem for our study, also deduced from funnel plots showing dubious asymmetry and resulting from small-study effects.

In addition, considering withdrawal due to AEs, we also included studies with no events in both treatment arms in the network. The Cochrane Handbook recommends omitting studies with rare events, but it is controversial whether this alters the bias evaluations and the accuracy of the combined estimation. Therefore, we included such trials and used a 0.5 zero-cell correction. However, we should explain these results with caution. It should be considered that though SUCRA is widely used to rank the effects of treatments, it may also ignore whether the difference between treatments has clinical significance. Although one treatment may be ranked as the best treatment, the absolute difference between the best one and other treatments may be very small. Therefore, also SUCRA results should be interpreted cautiously [15]. Finally, some factors can influence outcomes but cannot be adjusted or eliminated statistically. These include differences in comorbidities, duration and dose of treatment, time of follow-up and severity of psoriasis among populations in this study. For instance, some of the included studies have not reported the accurate duration or severity of psoriasis so that due to insufficient relevant data, it is almost impossible to adjust these factors.

## 5. Conclusion

Our research provides comprehensive comparative data for moderate to severe psoriasis patients in UV-based treatment. Our results revealed that the efficacy of all the combination therapy regimens was significantly superior to that of UV monotherapy, without significant differences in tolerability and safety. cUVB and cPUVA, and particularly the combination of UVA with calcium/vitamin D derivatives, was ranked as the overall safest and most effective phototherapy methods in UV-based treatment strategies. cPUVA and cUVB, especially combined with calcium/vitamin D derivatives, are both safe and effective treatments for psoriasis and have the potential for the first choice in the treatment of moderate-to-severe psoriasis. However, more high-quality trials are necessary for confirming our findings, and there will be detailed comparisons of relative efficacy and safety in future NMAs.

## Data Availability

Data generated or analysed during this study are included in this published article. The datasets generated during and/or analysed during this study are available from the corresponding author on reasonable request.

## Abbreviations

UV: Ultraviolet
PUVA: psoralen UVA
BB: broadband
NB: narrow-band
BB-UVB: Broadband UVB
NB-UVB: Narrowband UVB
RCT: randomized controlled trial
RR: risk ratio
PRISMA: Preferred Reporting Items for Systematic Reviews and Meta-Analyses
PASI: psoriasis area and severity index
AEs: adverse events
SUCRA: surface under the cumulative ranking
cPUVA: combined adjuvant therapy with PUVA
cAB: the combination of PUVA with UVB
PAVA: PUVA combined with vitamin A and its derivatives
PAVD: PUVA combined with vitamin D and its derivatives
UBVA: UVB combined with vitamin A and its derivatives
UBVD: UVB combined with vitamin D and its derivatives
UBST: UVB combined with systematic treatments
UBSL: UVB combined with skin lubricants
nRCTs: non-randomized control trials

## References

[1] Michalek IM, Loring B, John SM. A systematic review of worldwide epidemiology of psoriasis. J Eur Acad Dermatol Venereol. 2017;31(2):205–212. 27573025 10.1111/jdv.13854

[2] Griffiths CE, Christophers E, Barker JN, et al. A classification of psoriasis vulgaris according to phenotype. Br J Dermatol. 2007;156(2):258–262. 17223864 10.1111/j.1365-2133.2006.07675.x

[3] Elmets CA, Lim HW, Stoff B, et al. Joint American academy of Dermatology-National psoriasis foundation guidelines of care for the management and treatment of psoriasis with phototherapy. J Am Acad Dermatol. 2019;81(3):775–804. 31351884 10.1016/j.jaad.2019.04.042

[4] Vieyra-Garcia PA, Wolf PA. Deep dive into UV-based phototherapy: mechanisms of action and emerging molecular targets in inflammation and cancer. Pharmacol Ther. 2021;222:107784. 33316286 10.1016/j.pharmthera.2020.107784

[5] Leon A, Nguyen A, Letsinger J, et al. An attempt to formulate an evidence-based strategy in the management of moderate-to-severe psoriasis: a review of the efficacy and safety of biologics and prebiologic options. Expert Opin Pharmacother. 2007;8(5):617–632. 17376017 10.1517/14656566.8.5.617

[6] Menter A, Korman NJ, Elmets CA, et al. Guidelines of care for the management of psoriasis and psoriatic arthritis: section 5. Guidelines of care for the treatment of psoriasis with phototherapy and photochemotherapy. J Am Acad Dermatol. 2010;62(1):114–135. 19811850 10.1016/j.jaad.2009.08.026

[7] Almutawa F, Thalib L, Hekman D, et al. Efficacy of localized phototherapy and photodynamic therapy for psoriasis: a systematic review and Meta-analysis. Photodermatol Photoimmunol Photomed. 2015;31(1):5–14. 24283358 10.1111/phpp.12092

[8] Almutawa F, Alnomair N, Wang Y, et al. Systematic review of UV-based therapy for psoriasis. Am J Clin Dermatol. 2013;14(2):87–109. 23572293 10.1007/s40257-013-0015-y

[9] Mills EJ, Thorlund K, Ioannidis JP. Demystifying trial networks and network Meta-analysis. BMJ. 2013;346:f2914. 23674332 10.1136/bmj.f2914

[10] Schmitt J, Zhang Z, Wozel G, et al. Efficacy and tolerability of biologic and nonbiologic systemic treatments for moderate-to-severe psoriasis: meta-analysis of randomized controlled trials. Br J Dermatol. 2008;159(3):513–526. 18627372 10.1111/j.1365-2133.2008.08732.x

[11] Hampton P, Halliday A, Aassi M, et al. 12-week secukinumab treatment is consistently efficacious for moderate-to-severe psoriasis regardless of prior biologic and non-biologic systemic treatment: post-hoc analysis of six randomized trials. J Eur Acad Dermatol Venereol. 2021;35(4):928–937. 33030755 10.1111/jdv.16982 PMC7986672

[12] Brent MB, Brüel A, Thomsen JS. Animal models of disuse-induced bone loss: study protocol for a systematic review. Syst Rev. 2020;9(1):185. 32799918 10.1186/s13643-020-01441-3 PMC7429719

[13] Higgins JPT, Thomas J, Chandler J, et al (Eds.). Cochrane handbook for systematic reviews of interventions. 2nd ed. Chichester: John Wiley & Sons; 2019.

[14] White IR, Barrett JK, Jackson D, et al. Consistency and inconsistency in network Meta-analysis: model estimation using multivariate Meta-regression. Res Synth Methods. 2012;3(2):111–125. 26062085 10.1002/jrsm.1045 PMC4433771

[15] Rücker G, Schwarzer G. Ranking treatments in frequentist network Meta-analysis works without resampling methods. BMC Med Res Methodol. 2015;15:58. 26227148 10.1186/s12874-015-0060-8 PMC4521472

[16] Yones SS, Palmer RA, Garibaldinos TT, et al. Randomized double-blind trial of the treatment of chronic plaque psoriasis: efficacy of psoralen-UV-a therapy vs narrowband UV-B therapy. Arch Dermatol. 2006;142(7):836–842. 16847198 10.1001/archderm.142.7.836

[17] Dawe RS, Cameron H, Yule S, et al. A randomized controlled trial of narrowband ultraviolet B vs bath-psoralen plus ultraviolet a photochemotherapy for psoriasis. Br J Dermatol. 2003;148(6):1194–1204. 12828749 10.1046/j.1365-2133.2003.05482.x

[18] Lauharanta J, Juvakoski T, Lassus A. A clinical evaluation of the effects of an aromatic retinoid (tigason), combination of retinoid and PUVA, and PUVA alone in severe psoriasis. Br J Dermatol. 1981;104(3):325–332. 7011359 10.1111/j.1365-2133.1981.tb00957.x

[19] Torras H, Aliaga A, López-Estebaranz JL, et al. A combination therapy of calcipotriol cream and PUVA reduces the UVA dose and improves the response of psoriasis vulgaris. J Dermatolog Treat. 2004;15(2):98–103. 15204160 10.1080/09546630410023322

[20] Gordon PM, Diffey BL, Matthews JN, et al. A randomized comparison of narrow-band TL-01 phototherapy and PUVA photochemotherapy for psoriasis. J Am Acad Dermatol. 1999;41(5):728–732. 10534635 10.1016/s0190-9622(99)70008-3

[21] Ozdemir M, Engin B, Baysal I, et al. A randomized comparison of acitretin-narrow-band TL-01 phototherapy and acitretin-psoralen plus ultraviolet a for psoriasis. Acta Derm Venereol. 2008;88(6):589–593. 19002344 10.2340/00015555-0529

[22] Mortazavi H, Khezri S, Hosseini H, et al. A single blind randomized clinical study: the efficacy of isotretinoin plus narrow band ultraviolet B in the treatment of psoriasis vulgaris. Photodermatol Photoimmunol Photomed. 2011;27(3):159–161. 21535171 10.1111/j.1600-0781.2011.00581.x

[23] Dover JS, McEvoy MT, Rosen CF, et al. Are topical corticosteroids useful in phototherapy for psoriasis? J Am Acad Dermatol. 1989;20(5):748–754. 2654215 10.1016/s0190-9622(89)70085-2

[24] Ramsay CA, Schwartz BE, Lowson D, et al. Calcipotriol cream combined with twice weekly broad-band UVB phototherapy: a safe, effective and UVB-sparing antipsoriatric combination treatment. The Canadian calcipotriol and UVB study group. Dermatology. 2000;200(1):17–24. 10681608 10.1159/000018309

[25] Behrens S, Grundmann-Kollmann M, Schiener R, et al. Combination phototherapy of psoriasis with narrow-band UVB irradiation and topical tazarotene gel. J Am Acad Dermatol. 2000;42(3):493–495. 10688723 10.1016/s0190-9622(00)90225-1

[26] Dayal S, Mayanka, Jain VK. Comparative evaluation of NBUVB phototherapy and PUVA photochemotherapy in chronic plaque psoriasis. Indian J Dermatol Venereol Leprol. 2010;76(5):533–537. 20826993 10.4103/0378-6323.69081

[27] Al-Hamamy HR, Al-Mashhadani SA, Mustafa IN. Comparative study of the effect of narrowband ultraviolet B phototherapy plus methotrexate *vs.* narrowband ultraviolet B alone and methotrexate alone in the treatment of plaque-type psoriasis. Int J Dermatol. 2014;53(12):1531–1535. 24738793 10.1111/ijd.12444

[28] Ghiasi M, Ebrahimi S, Lajevardi V, et al. Efficacy and safety of pioglitazone plus phototherapy *versus* phototherapy in patients with plaque type psoriasis: a double blinded randomized controlled trial. J Dermatolog Treat. 2019;30(7):664–667. 30394148 10.1080/09546634.2018.1544702

[29] Bagel J. LCD plus NB-UVB reduces time to improvement of psoriasis *vs.* NB-UVB alone. J Drugs Dermatol. 2009;8(4):351–357. 19363853

[30] Brockow T, Schiener R, Franke A, et al. A pragmatic randomized controlled trial on the effectiveness of low concentrated saline spa water baths followed by ultraviolet B (UVB) compared to UVB only in moderate to severe psoriasis. J Eur Acad Dermatol Venerol. 2007;21(8):1027–1037. 10.1111/j.1468-3083.2007.02152.x 17714121

[31] Asawanonda P, Nateetongrungsak Y. Methotrexate plus narrowband UVB phototherapy *versus* narrowband UVB phototherapy alone in the treatment of plaque-type psoriasis: a randomized, placebo-controlled study. J Am Acad Dermatol. 2006;54(6):1013–1018. 16713455 10.1016/j.jaad.2006.01.004

[32] Mahajan R, Kaur I, Kanwar AJ. Methotrexate/narrowband UVB phototherapy combination *vs.* narrowband UVB phototherapy in the treatment of chronic plaque-type psoriasis–a randomized single-blinded placebo-controlled study. J Eur Acad Dermatol Venereol. 2010;24(5):595–600. 20015056 10.1111/j.1468-3083.2009.03486.x

[33] Calzavara-Pinton P. Narrow band UVB (311 nm) phototherapy and PUVA photochemotherapy: a combination. J Am Acad Dermatol. 1998;38(5 Pt 1):687–690. 9591811 10.1016/s0190-9622(98)70214-2

[34] Chauhan PS, Kaur I, Dogra S, et al. Narrowband ultraviolet B *versus* psoralen plus ultraviolet a therapy for severe plaque psoriasis: an Indian perspective. Clin Exp Dermatol. 2011;36(2):169–173. 20545955 10.1111/j.1365-2230.2010.03874.x

[35] Markham T, Rogers S, Collins P. Narrowband UV-B (TL-01) phototherapy vs oral 8-methoxypsoralen psoralen-UV-a for the treatment of chronic plaque psoriasis. Arch Dermatol. 2003;139(3):325–328. ]. 12622624 10.1001/archderm.139.3.325

[36] Brands S, Brakman M, Bos JD, et al. No additional effect of calcipotriol ointment on low-dose narrow-band UVB phototherapy in psoriasis. J Am Acad Dermatol. 1999;41(6):991–995. 10570386 10.1016/s0190-9622(99)70259-8

[37] Rim JH, Choe YB, Youn JI. Positive effect of using calcipotriol ointment with narrow-band ultraviolet B phototherapy in psoriatic patients. Photodermatol Photoimmunol Photomed. 2002;18(3):131–134. 12207676 10.1034/j.1600-0781.2002.180305.x

[38] Gahalaut P, Soodan PS, Mishra N, Rastogi MK, et al. Clinical efficacy of psoralen + sunlight *vs.* combination of isotretinoin and psoralen + sunlight for the treatment of chronic plaque-type psoriasis Vulgaris: a randomized hospital-based study. Photodermatol Photoimmunol Photomed. 2014;30(6):294–301. 24828298 10.1111/phpp.12125

[39] Menkes A, Stern RS, Arndt KA. Psoriasis treatment with suberythemogenic ultraviolet B radiation and a coal tar extract. J Am Acad Dermatol. 1985;12(1 Pt 1):21–25. 3980799 10.1016/s0190-9622(85)70003-5

[40] Saurat JH, Geiger JM, Amblard P, et al. Randomized double-blind multicenter study comparing acitretin-PUVA, etretinate-PUVA and placebo-PUVA in the treatment of severe psoriasis. Dermatologica. 1988;177(4):218–224. 2976000 10.1159/000248567

[41] Snellman E, Klimenko T, Rantanen T. Randomized half-side comparison of narrowband UVB and trimethylpsoralen bath plus UVA treatments for psoriasis. Acta Derm Venereol. 2004;84(2):132–137. 15206693 10.1080/00015550310022916

[42] Aggarwal P, Aggarwal K, Jain VK. Tacalcitol: a useful adjunct to narrow band ultraviolet B phototherapy in psoriasis. J Dermatolog Treat. 2016;27(6):546–551. 27052200 10.3109/09546634.2016.1163318

[43] Trott J, Gerber W, Hammes S, et al. The effectiveness of PUVA treatment in severe psoriasis is significantly increased by additional UV 308-nm excimer laser sessions. Eur J Dermatol. 2008;18(1):55–60. 18086590 10.1684/ejd.2008.0311

[44] Abdallah MA, El-Khateeb EA, Abdel-Rahman SH. The influence of psoriatic plaques pretreatment with crude coal tar *vs.* petrolatum on the efficacy of narrow-band ultraviolet B: a half-*vs.*-half intra-individual double-blinded comparative study. Photodermatol Photoimmunol Photomed. 2011;27(5):226–230. 21950625 10.1111/j.1600-0781.2011.00602.x

[45] Molin L. Topical calcipotriol combined with phototherapy for psoriasis. The results of two randomized trials and a review of the literature. Calcipotriol-UVB study group. Dermatology. 1999;198(4):375–381. 10490297 10.1159/000018151

[46] Neumann NJ, Mahnke N, Korpusik D, et al. Treatment of palmoplantar psoriasis with monochromatic excimer light (308-nm) *versus* cream PUVA. Acta Derm Venereol. 2006;86(1):22–24. 16585984 10.2340/00015555-0002

[47] Van TN, Van TH, Minh PPT, et al. Efficacy of Narrow - Band UVB phototherapy *versus* PUVA chemophototherapy for psoriasis in Vietnamese patients. Open Access Maced J Med Sci. 2019;7(2):227–230. 30745966 10.3889/oamjms.2019.057 PMC6364720

[48] Archier E, Devaux S, Castela E, et al. Efficacy of psoralen UV-a therapy *vs.* narrowband UV-B therapy in chronic plaque psoriasis: a systematic literature review. J Eur Acad Dermatol Venereol. 2012;26(3):11–21. 22512676 10.1111/j.1468-3083.2012.04519.x

[49] Gu X, Shen M, Zhao S, et al. Combination of targeted UVB phototherapy and calcipotriene *versus* targeted UVB alone in psoriasis: systematic review and Meta-analysis of randomized controlled trials. J Dermatolog Treat. 2020;1–5. 10.1080/09546634.2020.1770177 32419530

[50] Adachi Y, Uchida N, Matsuo T, et al. Clinical effect of vitamin D3 analogues is not inactivated by subsequent UV exposure. Photodermatol Photoimmunol Photomed. 2008;24(1):16–18. 18201352 10.1111/j.1600-0781.2008.00327.x

[51] Youn JI, Park BS, Chung JH, et al. Photoprotective effect of calcipotriol upon skin photoreaction to UVA and UVB. Photodermatol Photoimmunol Photomed. 1997;13(3):109–114. 9372528 10.1111/j.1600-0781.1997.tb00126.x

